# Supplementary material for: The VP53 protein encoded by RNA2 of a fabavirus, broad bean wilt virus 2, is essential for viral systemic infection
Source: Commun Biol. 2024 Apr 16;7:462. doi: 10.1038/s42003-024-06170-0 (PMC11021446; doi:10.1038/s42003-024-06170-0)
Supplement: Supplementary file 2 — Supplementary Information [file 42003_2024_6170_MOESM2_ESM.pdf]

Supplementary Figure S1

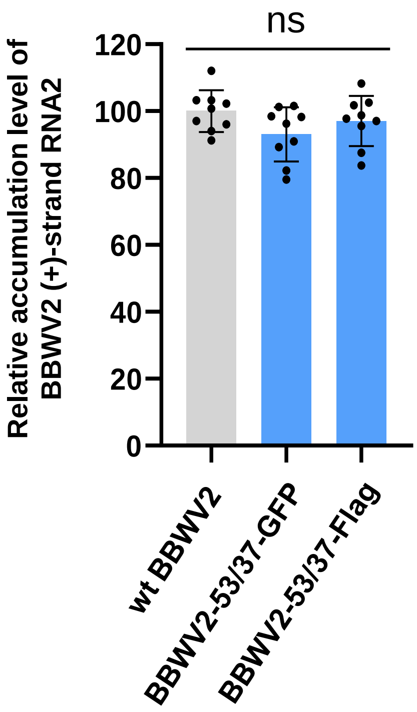

Fig. S1. Accumulation levels of BBWV2 recombinant viruses in systemically infected leaves. Total RNA was isolated from the upper symptomatic leaves of plants infected with wt BBWV2, BBWV2-53/37-GFP, or BBWV2-53/37-Flag at 10 dpi and subjected to RT-qPCR to analyze the relative accumulation levels of (+)-strand RNA2. The mean ± SD of three replications are shown and each column represents one group with nine plants. Significant differences were analyzed using a paired Student's t-test. ns = no significance (P>0.05).

Supplementary Figure S2

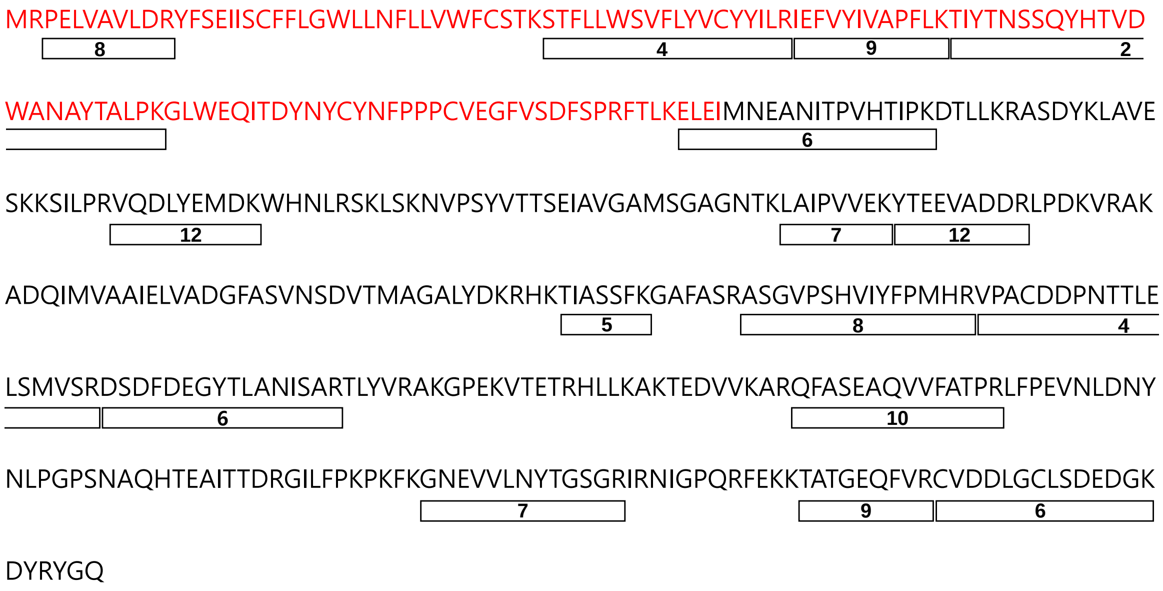

Fig. S2. Coverage map of the peptides obtained by LC-MS/MS analysis. A gel fragment corresponding to the molecular weight of VP53-Flag (~53 kDa) was excised from the SDS-PAGE gel, which separated the total protein extracted from the *N. benthamiana* leaves infected with BBWV2-53/37-Flag. The excised gel fragment was subjected to in-gel digestion using trypsin followed by LC-MS/MS analysis. The amino acid sequences of VP53 are shown. The red sequences highlight the N-terminal region of VP53, which does not overlap with VP37. The numbers within each box below the sequences indicate retention times for each peptide.

Supplementary Figure S3

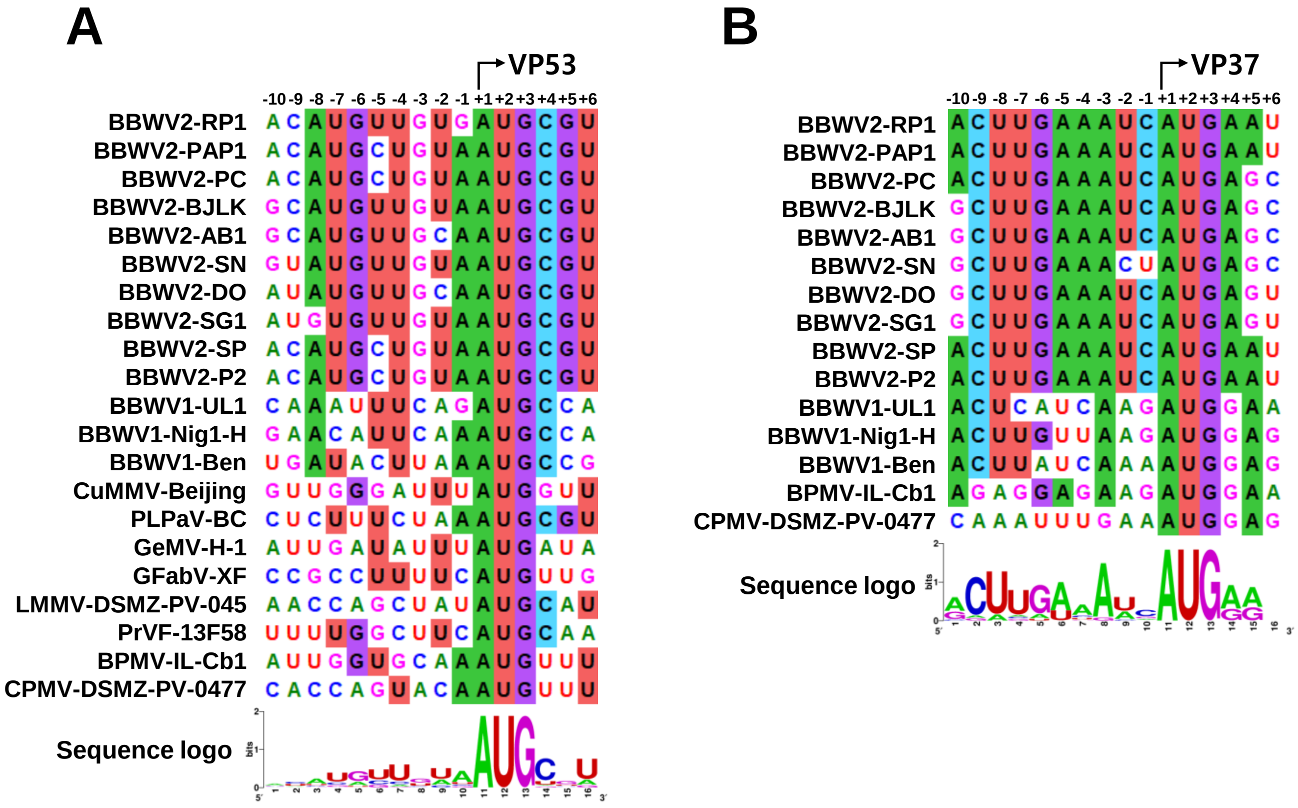

Fig. S3. Analysis of consensus sequences surrounding the AUG start codons for homologs of VP53 (A) and VP37 (B) among various members in the family *Secoviridae*. Consensus sequences were analyzed using ClustalW and illustrated using WebLogo, which presented stacks of symbols. The size of symbols within the stack reflects the relative frequency of each base at that position. For CuMMV, PLPaV, GeMV, GFabV, LMMV, and PrVF, the existence of a homolog for VP37 has not yet been elucidated. GenBank accession numbers: broad bean wilt virus 2 (BBWV2)-RP1 (KT380023), BBWV2-PAP1 (KT380021), BBWV2-PC (MW939477), BBWV2-BJLK (OP785726), BBWV2-AB1 (MH447989), BBWV2-SN (KX686590), BBWV2-DO (KT246496), BBWV2-SG1 (KJ789137), BBWV2-SP (KC625518), BBWV2-P2 (KC625512), broad bean wilt virus 1 (BBWV1)-UL1 (MF770980), BBWV1-Nig1-H (MN216379), BBWV1-Ben (KT988974), cucurbit mild mosaic virus (CuMMV)-Beijing (EU881937), peach leaf pitting-associated virus (PLPaV)-BC (MK460244), gentian mosaic virus (GeMV)-H-1 (AB084453), grapevine fabavirus (GFabV)-XF (MT086521), lamium mild mosaic virus (LMMV)-DSMZ-PV-045 (NC_023017), prunus virus F (PrVF)-13F58 (KX269875), bean pod mottle virus (BPMV)-IL-Cb1 (AY744933), and cowpea mosaic virus (CPMV)-DSMZ-PV-0477 (MT815985).

Supplementary Figure S4

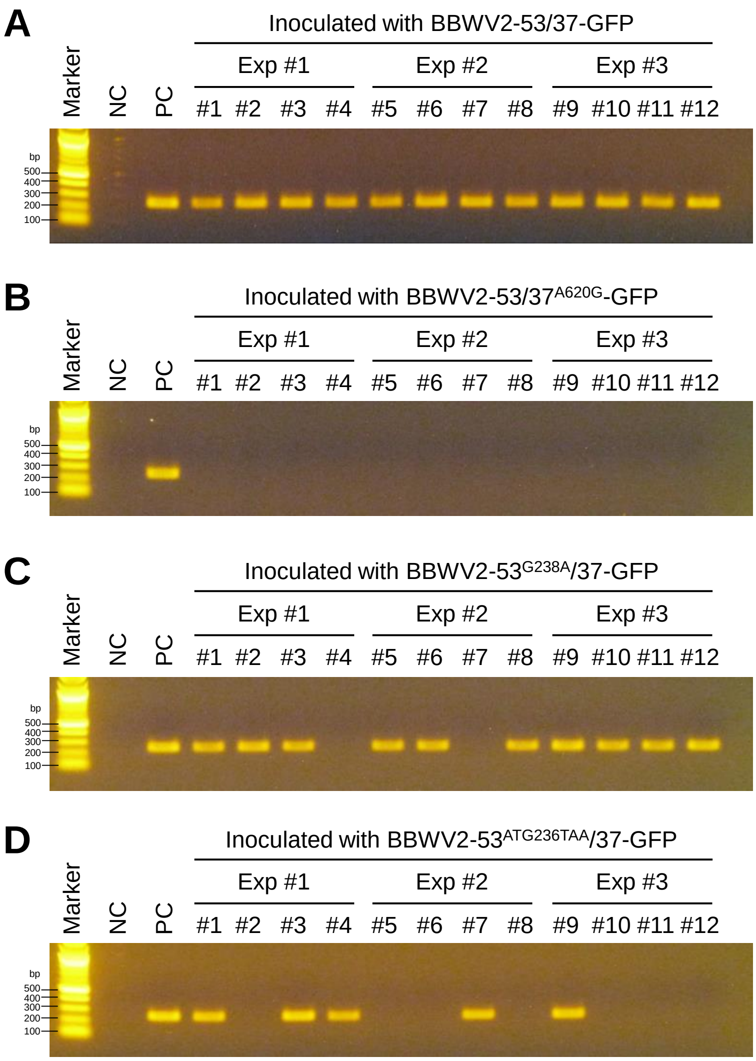

Fig. S4. RT-PCR detection of systemic infection of BBWV2 in *Nicotiana benthamiana* plants. Total RNA was extracted from the upper uninoculated leaves of *N. benthamiana* plants inoculated with either BBWV2-53/37-GFP (A), BBWV2-53/37^{A620G}-GFP (B), BBWV2-53^{G238A}/37-GFP (C), or BBWV2-53^{ATG236TAA}/37-GFP (D) 14 dpi and subjected to RT-PCR analysis using a BBWV2 -specific primer pair. Three independent experiments were performed, with each experiment involving the inoculation of four plants per viral construct. NC = negative control (total RNA from healthy *N. benthamiana* plants). PC = positive control (total RNA from *N. benthamiana* plants infected with wild-type BBWV2).

Supplementary Figure S5

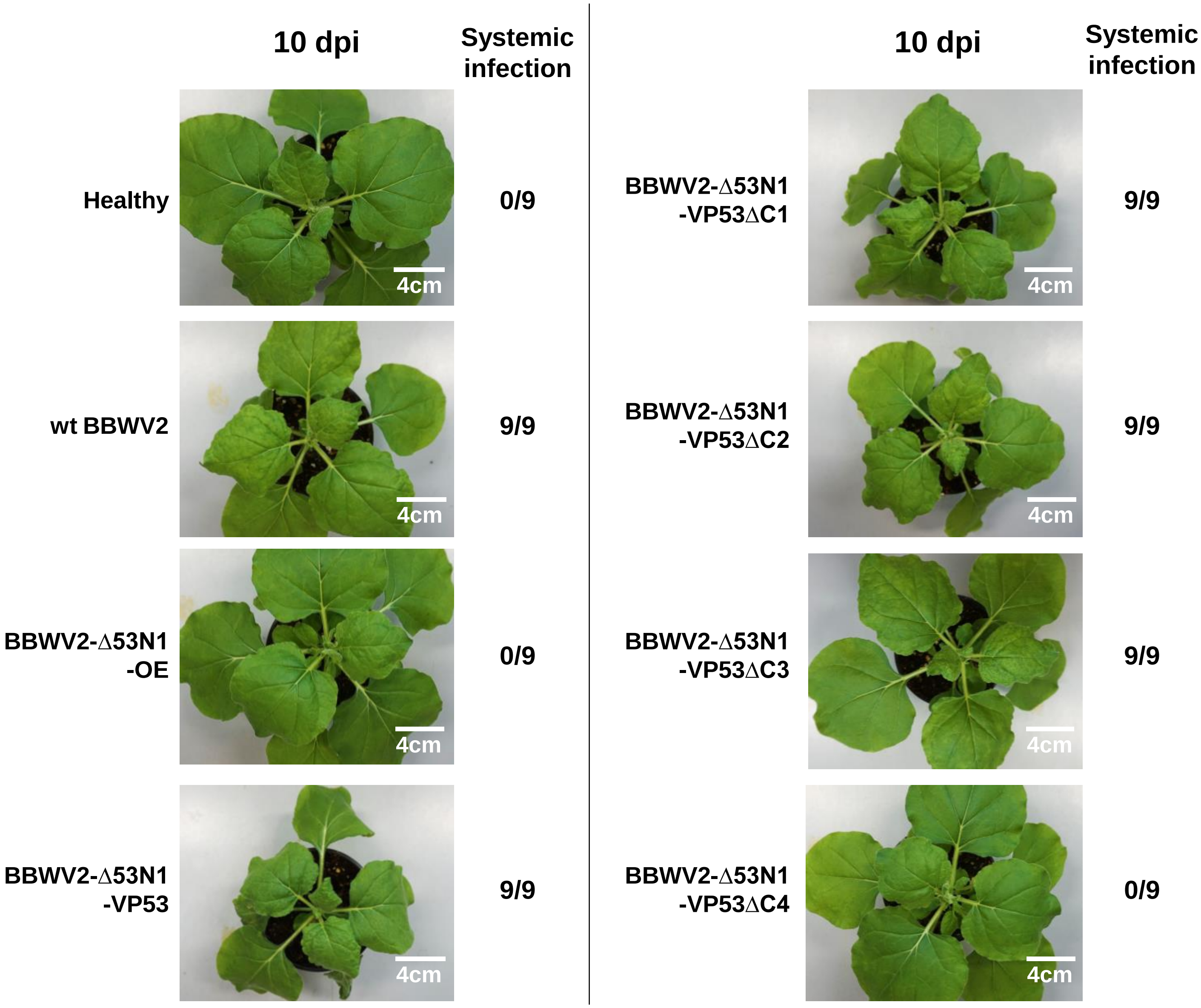

Fig. S5. Virulence of BBWV2-Δ53N1-OE, BBWV2-Δ53N1-VP53, BBWV2-Δ53N1-VP53ΔC1, BBWV2-Δ53N1-VP53ΔC2, BBWV2-Δ53N1-VP53ΔC3, and BBWV2-Δ53N1-VP53ΔC4 in *N. benthamiana*. *N. benthamiana* plants were inoculated with each BBWV2 recombinant viruses as indicated. Virus infection of the inoculated plants was determined by observing symptom development on the systemic leaves and confirmed by RT-PCR detection at 10 dpi. Data shown are representatives of three independent experiments, with each experiment involving the inoculation of three plants per viral construct. In total, nine plants were tested for the systemic infectivity of each virus (number of plants infected/number of plants inoculated).

Supplementary Figure S6

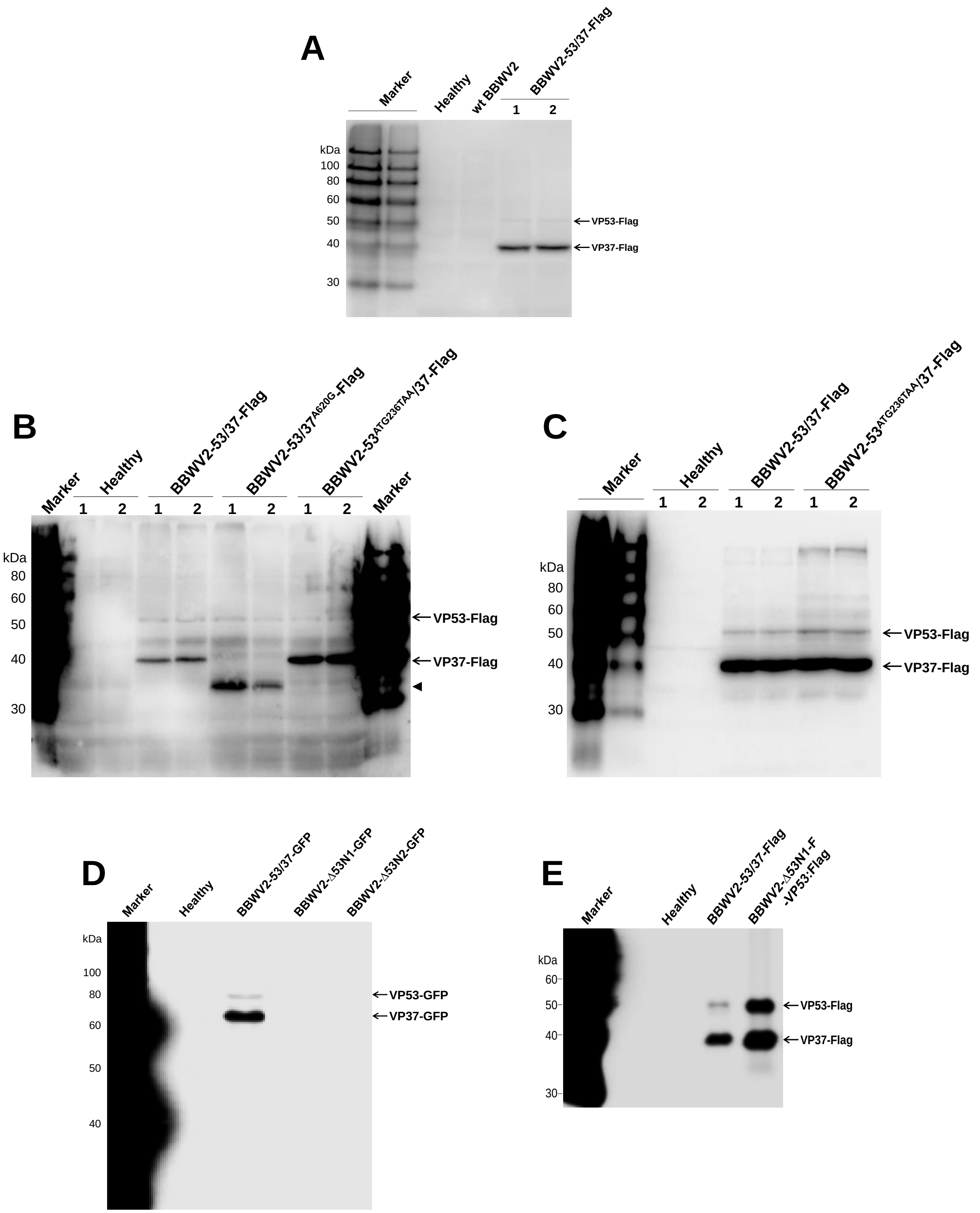

Fig. S6. Uncropped blot images for Figs. 3B (A), 5C (B), 5D (C), 7E (D), and 9C (E).

Supplementary Table S1. Primers used in this study.

Primer	Primer sequence (5' to 3')	Purpose
BBWV2R2-G238A-Fw	ACATGCTGTAATACGTCCCGAACTTGTTGCA	To introduce an G-to-A substitution at position 238 in RNA2
BBWV2R2-G238A-Rv	ACAAGTTCGGGACGTATTACAGCATGTTCA	
BBWV2R2-1AUGX-Fw	ACATGCTGTATAACGTCCCGAACTTGTTGCA	To introduce an ATG-to-TAA substitution at positions 236-238 in RNA2
BBWV2R2-1AUGX-Rv	ACAAGTTCGGGACGTTATACAGCATGTTCA	
BBWV2R2-A620C-Fw	GAACTTGAAATCCTGAATGAGGCAAATATCAC	To introduce an A-to-G substitution at nucleotide position 620 in RNA2
BBWV2R2-A620C-Rv	GATATTTGCCTCATTCAGGATTTCAAGTTCTTTAA	
BBWV2R2-5E-Fw	GTTTTAATAAAATATTAAAACAAACAGCTTTCG	To construct pBBWV2-R2-Δ53N1-GFP
Δ53N1-Fw	TCTTGAACATGCTGTAATGCGTCTGATTTCTCACCGAGAT	
Δ53N1-Rv	TGAACATGCTGTAATGCGTCTGATTTCTCACCGAGATTCA	
pBBWV2-R2-3E-KpnI-Rv	ATCCTCTAGAGGTACCTTTTTTTT	
BBWV2R2-5E-Fw	GTTTTAATAAAATATTAAAACAAACAGCTTTCG	To construct pBBWV2-R2-Δ53N2-GFP
Δ53N2-Fw	CTTGAACATGCTGTAATGAATGAGGCAAATATCACTC	
Δ53N2-Rv	ATATTTGCCTCATTCATTACAGCATGTTCAAGATCAA	
pBBWV2-R2-3E-KpnI-Rv	ATCCTCTAGAGGTACCTTTTTTTT	
VP53-BglII-Fw	GAAGATCTATGCGTCCCGAACTTGTTGCA	To amplify the full-length VP53 or its C-terminal deletions
VP53-FL-AvrII-Rv	GATCCTAGGTTGACCATATCTATAATCTTTGCCA	
VP53-298-AvrII-Rv	GATCCTAGGTGCAGGGACTCTATGCATTG	
VP53-128-AvrII-Rv	GATCCTAGGGATTTCAAGTTCTTTAAGTGTGAA	
VP53-84-AvrII-Rv	GATCCTAGGCGCGTTTGCCCAATCTACAG	
VP53-64-AvrII-Rv	GATCCTAGGGGGTGCAACGATATACACAAAT	
VP53-BglII-Fw	GAAGATCTATGCGTCCCGAACTTGTTGCA	To amplify the full-length VP53 containing a Flag epitope at the C-terminus
VP53-Flag-AvrII-Rv	GATCCTAGGCTTGTCATCGTCGTCCTTGTAG	
VP53-Fw	ATGCGTCCCGAACTTGTTGCA	To construct PZP-VP53-GFP and PZP-VP37-GFP
VP37-Fw	ATGAATGAGGCAAATATCACTCC	
VP53/37-SpeI-Rv	GGACTAGTTTGACCATATCTATAATCTTTGC	
